# Editorial: Microbial Responses to Environmental Changes

**DOI:** 10.3389/fmicb.2015.01364

**Published:** 2015-12-02

**Authors:** Jürg B. Logue, Stuart E. G. Findlay, Jérôme Comte

**Affiliations:** ^1^Aquatic Ecology, Department of Biology, Lund UniversityLund, Sweden; ^2^Science for Life LaboratoryStockholm, Sweden; ^3^Cary Institute of Ecosystem StudiesMillbrook, NY, USA; ^4^Département de Biologie, Centre d'études Nordiques - Takuvik and Institut de Biologie Intégrative et des Systèmes, Université LavalQuébec, QC, Canada

**Keywords:** ecosystem functioning, environmental change, microbial community composition, microbial diversity, microbial ecology, micro-organism, next-generation sequencing

Earth is teeming with taxonomically, phylogenetically, and metabolically highly diverse micro-organisms; organisms that are crucial for sustaining life on our planet in that they carry out processes of great biogeochemical significance. The processes and mechanisms underlying their diversity and distribution are, thus, of great interest, yet—despite their importance—only poorly understood.

Microbial communities may be structured by a large number of factors among which are environmental conditions: local, contemporary environmental conditions select and sort micro-organisms according to their ecological niche (Hutchinson, 1957). This corresponds to the so-called Baas-Becking hypothesis for microbial organisms “*everything is everywhere:* but *the environment selects*” (Baas Becking, 1934) that formed the basis of the later formulated species-sorting paradigm/perspective (Leibold et al., 2004; Holyoak et al., 2005). Species sorting has been repeatedly shown to be an important determinant for the assembly of microbial communities (e.g., Van der Gucht et al., 2007; Logue and Lindström, 2008). It has been suggested that the response of microbes to environmental conditions and changes thereof is mediated by a complex combination of adjustment (e.g., high adaptability and plasticity but also ability to horizontally transfer genetic material), replacement (e.g., high dispersal rates) and species interaction mechanisms, all facilitated by their fast population growth rates (Allison and Martiny, 2008; Comte and del Giorgio, 2011). Yet, the factors that determine the type and magnitude of the response of microbial communities remain unclear (de Vries and Shade, 2013). Given that many ecosystems are undergoing rapid and major environmental changes, obtaining a quantitative and process-level understanding of the mechanisms that affect microbes and microbial communities is pivotal for predicting the responses of microbial communities to novel or changing selective forces and their implications at the local, regional and global scale.

Advances in molecular biology have revolutionized our ability to describe microbial communities with regard to composition and biogeography, diversity and processing, but also the mechanisms of adaptation and evolution (Pedrós-Alió, 2006). In addition, the integration of theory into microbial ecology has greatly improved our understanding of the roles micro-organisms play across a wide range of biomes by providing researchers with organization, structure, mechanistic insight, and predictive power (Prosser et al., 2007). Technical advances and theoretical integration will advance our understanding of how microbial organisms may respond and evolve in a changing environment.

The aim of this Research Topic “Microbial responses to environmental changes” is to provide the reader with a selection of studies that have gone beyond a descriptive level and investigated the mechanisms by which microbial communities and associated processes respond to environmental gradients and changes. This Research Topic brings together studies that applied the latest molecular techniques for studying the composition and functioning of microbial communities and integrated ecological, biogeochemical and/or modeling approaches to provide a comprehensive and mechanistic perspective of the responses of micro-organisms to environmental changes. Contributions include original research (i.e., field surveys and laboratory or field experiments) and comprehensive reviews. They outline critical challenges that remain as well as new perspectives and ideas with regard to responses of micro-organisms to changes in the environment. Thus, patterns of response of microbial communities to environmental gradients and changes are documented for archaea, bacteria, fungi, and microbial eukaryotes, as well as a vast range of habitats including streams, ponds and lakes, ocean and seas, and sediments and soils, and varying spatial and temporal scales.

This Research Topic presents new findings on environmental variables influencing microbial communities, the type and magnitude of responses and differences in the response among microbial groups. As such, several studies have focused on how environmental drivers affected by climate change can structure microbial communities (Koyama et al., 2014; DeAngelis et al., 2015; Wu et al., 2015) and regulate microbial activities by, for example, influencing production (Aanderud et al., 2015; Adams et al., 2015), respiration (Amend et al., 2015), activity of enzymes (Berlemont et al., 2014; Penton et al., 2015), or carbon transformations and sequestration (Liang et al., 2015). Environmental field surveys, on the other hand, identified shifts in microbial community composition due to a range of environmental variables (Crevecoeur et al., 2015; Febria et al., 2015; Ling et al., 2015; Nguyen and Landfald, 2015; Sarmento et al., 2015).

To examine the type of response, experimental studies identified both adjustment and replacement effects, where microbial communities either adapted or shifted in composition as a result of environmental drivers. Aanderud et al. (2015) identified hundreds of rare bacterial taxa that increased in abundance within only a few days after rewetting of dry soil, some becoming even dominant and contributing to ecosystem functioning. DeAngelis et al. (2015), on the other hand, observed that soil microbial communities at first adapted to warming conditions before eventually (i.e., after 20 years) truly shifting in composition. In a short-term transplant experiment, Lindh et al. (2015) demonstrated that the mechanisms underlying the response of bacterial communities to changes in the environmental conditions varied depending on the successional state during which the change happened. This highlights the importance of considering time, when investigating the nature of response to changes in the environment.

A central question in ecology is how community composition influences ecosystem functioning (Loreau et al., 2002). Microbial ecologists, in particular, are faced with not fully comprehending whether changes in community composition lead to shifts in microbially mediated functions or whether shifts in these functions require changes in composition. Understanding is yet further hampered by the notion that environmental forces drive changes in both composition and function. Previous work has illustrated that exploring functional properties (i.e., traits) in this respect is highly promising (e.g., Martiny et al., 2015; Ruiz-Conzález et al., 2015) and that the distribution of specific traits may govern the type of microbial response to environmental changes (e.g., Shade and Handelsman, 2012). A number of contributions explored how microbial assemblages responded to changes in environmental variables via trait variation. Mackey et al. (2014) analyzed the effects of changes in the iron-nitrogen ratio on phytoplankton and observed that nitrate additions favored slow-sinking single-celled over faster sinking chain-forming diatoms. Sarmento et al. (2015) showed that microbial life in aquatic surface microlayers was governed by different independent processes compared to that in the underlying waters. It is assumed that these surface microlayer communities directed most of their energy toward maintenance and repair processes due to the high UV-radiation levels rather than growth efficiency, which was higher in deeper layers. Changes have also been reported at the cell level, where heterotrophic bacteria featuring a range of homeostatic regulation mechanisms were able to accommodate changes in carbon and phosphorus concentrations (Godwin and Cotner, 2015). Crowther et al. (2014) have, in this respect, proposed a conceptual trait-based framework for studying how niche processes, such as stress tolerance or combative dominance, structure fungal assemblages across time and space, which in turn may provide insight into the relationship between community composition and functioning.

A major challenge in microbial ecology is to determine whether micro-organisms show unique features or have patterns in common with macro-organisms (i.e., plants and animals). Yet, whether distinct patterns can be distinguished between the different micro-organisms has received far less attention. In other words, do bacteria, for example, respond in the same way to environmental cues as archaea, fungi or microbial eukaryotes? Given that intrinsic properties but also key variables and processes involved in structuring the respective communities are different from each other, it is highly unlikely that responses follow the same patterns among these groups of micro-organisms. Indeed, several studies revealed distinct patterns by which, for instance, archaea and bacteria or bacteria and fungi responded to changes in their environment or particular environmental stressors. Nguyen and Landfald (2015), having examined variation in archaeal and bacterial community composition over a moderate environmental gradient naturally existing in the Barents Sea, showed that archaeal assemblages appeared to be structured by one specific variable (i.e., level of freshly sedimented phytopigments), while bacterial communities were significantly influenced by a broader set of environmental variables. Contributions focusing on bacteria and fungi highlighted differences in the sensitivity to drought (Berlemont et al., 2014), fertilization (Koyama et al., 2014; Liang et al., 2015) and warming (DeAngelis et al., 2015; Liang et al., 2015), which in turn impacted measured functional processes or traits (Berlemont et al., 2014; Liang et al., 2015). Finally, the sensitivity or resilience of diverse aquatic microbiota to environmental variation assessed in a review and synthesis analysis by Zeglin (2015) demonstrated that bacterial communities reacted differently to various environmental drivers compared to other microbial groups. Thus, assemblages may tend to converge or diverge as particular drivers wax or wane.

In summary, with ongoing climate change and alteration of habitats' integrity and structure, a mechanistic understanding of the implications in terms of ecosystem functioning is greatly needed. The broad-range of articles presented here deepen our current understanding and knowledge of the underlying mechanisms of microbial structural and functional responses to environmental changes and gradients in both aquatic and terrestrial ecosystems. The body of work has, furthermore, identified many challenges and questions that remain to be addressed and new perspectives to follow up on. We are hopeful that this collection of studies will stimulate discussions and pave the way for future prospects far beyond this Research Topic.

## Author contributions

All authors contributed to the writing of the editorial.

## Funding

This Research Topic would not have been realized without the support of the Swiss National Science Foundation (SNSF; #PA00P3_145355, supporting JBL), the Natural Sciences and Engineering Research Council of Canada (NSERC) via the CREATE training program in northern environmental sciences (EnviroNorth; supporting JC), and the Fonds de recherche du Québec—Nature et technologies (FRQNT; #165244, supporting JC).

### Conflict of interest statement

The authors declare that the research was conducted in the absence of any commercial or financial relationships that could be construed as a potential conflict of interest.
